# Response to Letter to the Editor “Atrial Fibrillation and Heart Failure: Synergistic Effect on Functional Class and Quality of Life”

**DOI:** 10.1002/clc.70159

**Published:** 2025-06-04

**Authors:** James Samír Díaz, Johanna Marcela Vanegas

**Affiliations:** ^1^ Clínica Las Américas Auna Medellín Colombia; ^2^ School of Health Sciences Universidad Pontificia Bolivariana Medellín Colombia

**Keywords:** atrial fibrillation, functional class, heart failure, left ventricular ejection fraction, prognosis, quality of life

Response to Editor,

We welcome and appreciate the comments raised by Kataoka and colleagues related to our recent publication “Atrial fibrillation and heart failure: synergistic effect on functional class and quality of life” [1]. In this study, we compared the evolution of functional class and quality of life in patients with heart failure (HF) according to the presence of atrial fibrillation (AF). The results highlighted the significant impact of AF on functional status in patients with HF. The coexistence of AF and reduced ejection fraction primarily impaired the physical dimension of quality of life (QoL) and limited improvement in NYHA functional class, underscoring the need for targeted management of these conditions in comprehensive HF care.

We appreciate the opportunity to clarify key aspects of our study and to address the important points raised regarding patient selection, AF subtypes, diagnostic definitions, and therapeutic strategies. These insights have allowed us to enhance the clarity and clinical relevance of our work. Below, we provide detailed, point‐by‐point responses to each of the comments.

In our study, we included patients across the full spectrum of LVEF, without applying exclusions based on LVEF range or age. This decision reflects the real‐world population typically seen in HF clinics and was necessary given the relatively small sample size. To mitigate potential confounding, we performed stratified subgroup analyses according to LVEF (≤ 40% vs. > 40%). We agree that a larger study, with more narrowly defined LVEF categories, could provide greater statistical power to better delineate the interaction between AF and systolic function in patient‐reported outcomes [2].

Regarding the absence of distinction between paroxysmal and persistent AF, we acknowledge the importance of differentiating AF subtypes when evaluating its impact on QoL. Unfortunately, due to the retrospective nature of our study and limitations in the available clinical data, we were unable to consistently classify AF as paroxysmal or persistent/permanent across all patients. It is also important to note that our study focused on patients with coexisting AF and chronic HF, rather than AF in isolation, where the type of AF may have a more direct impact on patient QoL.

We agree that future prospective studies should include stratification by AF subtype and arrhythmia burden to more accurately assess their differential effects on clinical outcomes. Given the retrospective nature of our study, we defined arrhythmia‐induced cardiomyopathy as cases of HF with coexisting AF in which LVEF improved following rhythm control or rate optimization. We agree that this entity may overlap with idiopathic cardiomyopathy in terms of clinical presentation. However, in our HF program, patients who do not show improvement in LVEF after achieving adequate ventricular rate control are routinely undergo further studies, including cardiac magnetic resonance to look for other causes of cardiomyopathy.

Thank you for highlighting the point regarding rhythm versus rate control strategies. All patients in our cohort were managed according to contemporary HF guidelines, with rhythm or rate control strategies individualized based on comorbidities, symptom burden, and input from electrophysiology specialists [3, 4]. Pharmacological management was the predominant approach, and catheter ablation was selectively employed based on specific indications. The observed overall improvement in NYHA functional class likely reflects the benefits of comprehensive, multidisciplinary care, which included optimal medical therapy, device‐based interventions when appropriate, and lifestyle modification support.

It is also worth noting that our study included patients enrolled between 2020 and 2022, a period during which routine AF ablation in patients with HF had not yet been widely adopted in clinical practice. With the publication of the CASTLE‐HTx trial in late 2023, routine ablation may now be more frequently considered for this population, and future research will be needed to assess its real‐world impact [5].

## Data Availability

The data that support the findings of this study are available from the corresponding author upon reasonable request.
